# REV7 is essential for DNA damage tolerance via two REV3L binding sites in mammalian DNA polymerase ζ

**DOI:** 10.1093/nar/gku1385

**Published:** 2015-01-07

**Authors:** Junya Tomida, Kei-ichi Takata, Sabine S. Lange, Andria C. Schibler, Matthew J. Yousefzadeh, Sarita Bhetawal, Sharon Y.R. Dent, Richard D. Wood

**Affiliations:** 1 Department of Epigenetics and Molecular Carcinogenesis, The University of Texas MD Anderson Cancer Center Science Park, Smithville, TX 78957, USA; 2 The University of Texas Graduate School of Biomedical Sciences at Houston, Houston, TX 77030, USA

## Abstract

DNA polymerase zeta (pol ζ) is exceptionally important for controlling mutagenesis and genetic instability. REV3L comprises the catalytic subunit, while REV7 (MAD2L2) is considered an accessory subunit. However, it has not been established that the role of REV7 in DNA damage tolerance is necessarily connected with mammalian pol ζ, and there is accumulating evidence that REV7 and REV3L have independent functions. Analysis of pol ζ has been hampered by difficulties in expression of REV3L in mammalian cells, and lack of a functional complementation system. Here, we report that REV7 interacts with full-length REV3L *in vivo* and we identify a new conserved REV7 interaction site in human REV3L (residues 1993–2003), distinct from the known binding site (residues 1877–1887). Mutation of both REV7-binding sites eliminates the REV3L–REV7 interaction. *In* *vivo* complementation shows that both REV7-binding sites in REV3L are necessary for preventing spontaneous chromosome breaks and conferring resistance to UV radiation and cisplatin. This demonstrates a damage-specific function of REV7 in pol ζ, in contrast to the distinct roles of REV3L and REV7 in primary cell viability and embryogenesis.

## INTRODUCTION

Although many types of DNA damage cause replication forks to stall temporarily, the DNA polymerases used for semi-conservative replication generally cannot proceed on damaged DNA (1,2). A base lesion may be bypassed, however, by invoking a process of translesion DNA synthesis (TLS) mediated by specialized DNA polymerases. In mammalian cells, TLS can take place at the DNA replication fork, or at post-replication gaps containing a lesion (3,4). DNA polymerase zeta (pol ζ) is of central importance for TLS in eukaryotes. In mammalian cells it is needed for the bypass of many DNA lesions, even though pol ζ-mediated TLS can be mutagenic if an incorrect base is inserted opposite a mis-instructional lesion in the DNA template (4–6). If pol ζ-mediated TLS is not accomplished in a timely manner, the machinery at the DNA replication fork can collapse, and subsequent enzymatic action can cut the DNA at the non-functional replication fork and form a double-strand break. Suppression of pol ζ-mediated TLS can sensitize tumors to chemotherapy and reduce the frequency of acquired drug resistance (7). In addition to dealing with DNA damaged by environmental agents, pol ζ aids in replication of some naturally occurring DNA sequences that are inherently difficult to traverse, such as the ‘fragile-site’ regions in mammalian genomes or sequences forming non-B DNA structures (8,9). In pol ζ-defective mammalian cells, DNA double-strand breaks form in proliferating cells, with ensuing chromosomal rearrangements (10–13).

The biology and biochemistry of pol ζ was examined first in the yeast *Saccharomyces cerevisiae*, where the catalytic subunit is encoded by the *REV3* gene. Yeast Rev3 is associated with a protein encoded by the *REV7* gene. In mammalian cells, a homologous catalytic subunit is encoded by the *REV3L* gene. The REV3L protein of ∼3100 amino acid residues in human and mouse cells is about twice the size of the yeast protein. The closest Rev7 protein homolog in mammalian cells is called REV7 (gene *MAD2L2*).

By primary protein sequence similarity, Rev3 and REV3L are assigned as ‘B’ family DNA polymerases; the other members of this family in yeast and mammalian cells are the catalytic subunits of the DNA polymerases involved in chromosomal replication. In mammalian cells these are POLA1, POLD1 and POLE, the catalytic subunits of pol α, pol δ and pol ϵ respectively. Each of these B-family catalytic subunits has a conserved [4Fe-4S] iron-sulfur cluster near the C-terminus that serves as a docking site for additional subunits. For yeast Rev3, these subunits are Pol31 and Pol32*;* in mammalian cells, the orthologous subunits are designated POLD2 and POLD3 (14–17). These proteins are also subunits of the replicative DNA polymerase δ. The shared association of the catalytic subunits of pol δ and pol ζ with these auxiliary subunits may provide a mechanism for the two polymerases to switch places when normal DNA replication is stalled at a template DNA lesion (14,18).

In contrast, the specific function of the mammalian REV7 protein within pol ζ is less clear. REV7 is unusual because it does not have a counterpart in the other B family DNA polymerases. An overall view of 4-subunit yeast pol ζ, obtained by electron microscopy shows that REV7 contacts the central region of REV3 and seems unlikely to make direct contacts with DNA (19). Mammalian REV7 participates in DNA damage resistance functions but it is unclear whether it does so only as a subunit of pol ζ (20), or whether REV7 has pol ζ-independent functions that are more important. Rev7 does appear to be important for yeast pol ζ activity; although Rev3 has some DNA polymerase activity on its own, the activity is greatly stimulated in a complex with Rev7 (21). Human REV7 is bound tightly enough to REV3L so that pol ζ can be purified by virtue of an affinity tag on REV7, but the specific effect of REV7 omission has not been tested. Using extracts from *Drosophila* cells, insect REV3 was purified following binding to an affinity chromatography column charged with REV1. The REV7 subunit was not apparent in this preparation, and the addition of tagged REV7 protein did not stimulate purified *Drosophila* pol ζ (22). Further, as discussed more extensively below, REV7 is much more abundant than REV3L and knockout mice for the two genes have strikingly different phenotypes.

To determine whether REV7 is necessary for the DNA damage tolerance function of mammalian pol ζ, we analyzed REV3L mutants unable to bind REV7 using a newly developed functional complementation system. During this process we located a previously unidentified interaction site for REV7 located in human REV3L. We show that both binding sites are needed to maintain chromosomal stability and resistance to DNA damage.

## MATERIALS AND METHODS

### Human cell cultures and transfections

Human 293T cells were maintained in Dulbecco's Modified Eagle's medium GlutaMAX™ (Life Technologies, Carlsbad, CA, USA) supplemented with 10% fetal bovine serum and penicillin/streptomycin in a 5% CO_2_ incubator at 37°C. Human HeLa S3 cells were maintained in RPMI-1640 medium supplemented with 10% fetal bovine serum and penicillin/streptomycin in a 5% CO_2_ incubator at 37°C. Human FH-REV3L-expression vector was transfected into 293T and control cells using Lipofectamine 2000 (Life Technologies) according to the manufacturer's instructions. All cell lines were routinely checked for mycoplasma contamination using the MycoAlert detection kit (Lonza). Cell lines were validated by short tandem repeat (STR) DNA fingerprinting by the Cell Line Identification Core of the MD Anderson Cancer Center. The STR profiles were compared to known ATCC fingerprints (ATCC.org), to the Cell Line Integrated Molecular Authentication database (CLIMA) version 0.1.200808 (http://bioinformatics.istge.it/clima/) (23) and to the MD Anderson cell line fingerprint database.

### Antibodies

We used the following antibodies: ProteinTech (polyclonal anti-REV7/MAD2L2 1:1200; 12683-1-AP), Sigma-Aldrich (polyclonal anti-FLAG 1:1000; F7425, monoclonal anti-αTubulin 1:8000; T5168, HRP (horseradish peroxidase) conjugated anti-mouse IgG 1:10 000; A0168, HRP conjugated anti-rabbit IgG 1:10 000; A0545, anti-FLAG M2 affinity agarose gel: A2220), Clontech (polyclonal anti-GFP 1: 3000; 632460, 632592), Cell Signaling Technology (monoclonal anti-HA (C29F4) 1:1000; #3724, monoclonal anti-His (27E8) 1:1000; #2366), Abcam (polyclonal anti-Histone H3 1:5000; ab1791), GE Healthcare (anti-GST-HRP 1:8000; RPN1236), MBL (agarose conjugated anti-GFP (RQ2); D153-8)

### DNA constructs and shRNA oligonucleotides

The human *REV3L* full-length cDNA was obtained in the pUC19M1 vector from Zhigang Wang (24) and subcloned by Dr Gregory Gan. The full length wild-type (WT) gene was polymerase chain reaction (PCR) amplified from REV3L cDNA as a XhoI–NotI fragment with 5′REV3L (XhoI) primer (5′-CCGCTCGAGATGTTTTCAGTAAGGATAGTGAC) and 3′REV3L (NotI) primer (5′-TAAAAGCGGCCGCTTAAAACTGGTCTAATAACTGCCGG) to clone into pETDuet-1 (Novagen). REV3L (P1880A, P1885A), REV3L (P1996A, P2001A) and REV3L (P1880A, P1885A, P1996A, P2001A)/pETDuet-1 mutations were introduced using site-directed mutagenesis kits (Stratagene). The XhoI–NotI fragments from WT REV3L, REV3L (P1880A, P1885A), REV3L (P1996A, P2001A) and REV3L (P1880A, P1885A, P1996A, P2001A)/pETDuet-1 were inserted into pOZN (25) (kindly provided by Hank Heng Qi, Children's Hospital, Boston, MA, USA). pCDH-EF1α-Flag-HA-MCS-IRES-Puro was PCR amplified from pOZN as a EcoRI fragment with 5′ EcoRI-FLAG-HA primer (5′-GGAATTCATGGACTACAAGGACGACGATGACAAG) and 3′pOZ primer (5′-CGGAATTGATCCGCTAGAG) to clone into pCDH-EF1α-MCS-IRES-Puro (System Biosciences). The XhoI–NotI fragments from WT REV3L or REV3L (P1880A, P1885A), REV3L (P1996A, P2001A) and REV3L (P1880A, P1885A, P1996A, P2001A)/pETDuet-1 were inserted into pCDH-EF1α-Flag-HA-MCS-IRES-Puro to generate FH-REV3L, FH-REV3L (P1880A, P1885A), FH-REV3L (P1996A, P2001A) and FH-REV3L (P1880A, P1885A, P1996A, P2001A).

FH-REV3L 1974–3130 and 2000–3130 fragments were PCR amplified from REV3L/pOZN as a XhoI–NotI fragment with 5′REV3L 1974 (XhoI) primer (5′-CCGCTCGAGGGAGTTGTCAATAAAGGGTCAAG) or 5′REV3L 2000 (XhoI) primer (5′-CCGCTCGAGGCCCCAAGTCGACAACTGGTTC) and 3′pOZ primer to clone into pCDH-EF1α-Flag-HA-MCS-IRES-Puro. GFP-REV3L 1974–1999, 1974–2025 and 1974–2075 expression vector were PCR amplified from REV3L/pETDuet-1 as a XhoI–BamHI fragment with 5′REV3L 1974 (XhoI) primer and 3′ REV3L 1999 (BamHI) primer (5′-CGGGATCCTTAACATTTGCAAGGCATAATCAC), 3′ REV3L 2025 (BamHI) primer (5′-CGGGATCCTTAGGTTTTAGGCAGTTTCTTGGAAC) or 3′ REV3L 2075 (BamHI) primer (5′-CGGGATCCTTAAATCTGAGAATTATCAACATCC) to clone into pEGFP-C1 (Clontech). REV3L 1974–2025 (P1996A, P2001A)/pEGFP-C1 mutations were introduced using site-directed mutagenesis kits. GST-REV3L 1974–1999 and 1974–2025 fragments were PCR amplified from REV3L/pETDuet-1 as a XhoI–NotI fragment with 5′REV3L 1974 (XhoI) primer and 3′ REV3L 1999 (NotI) primer (5′-TAAAAGCGGCCGCTTAACATTTGCAAGGCATAATCAC) or 3′ REV3L 2025 (NotI) primer (5′-TAAAAGCGGCCGCTTAGGTTTTAGGCAGTTTCTTGGAAC) to clone into pGEX6P-1 (GE Healthcare). REV3L 1974–2025 (P1996A, P2001A)/pGEX6P-1 mutations were introduced using site-directed mutagenesis kits. GST-REV3L 1847–1898 fragment was PCR amplified from REV3L/pETDuet-1 as a XhoI–NotI fragment with 5′REV3L 1847 (XhoI) primer (5′-CCGCTCGAGATGTTGACACCAACTCCTGATAGTTC) and 3′ REV3L 1898 (NotI) primer (5′-TAAAAGCGGCCGCTTAGTCATGATCCAACAAAGTTGCC) to clone into pGEX6P-1. His-REV7 expression vector was PCR amplified from REV7 cDNA as a XhoI–NotI fragment with 5′REV7 (XhoI) primer (5′-CCGCTCGAGATGACCACGCTCACACGACAAG) and 3′ REV7 (NotI) primer (5′-TAAAAGCGGCCGCTCAGCTGCCTTTATGAGCGCG) to clone into pETDuet-1 (SalI–NotI) (Novagen). After construction, all expression vectors were confirmed by DNA sequencing. The shREV7 was purchased from Open Biosystems. The shScramble RNA (Plasmid 1864) was purchased from Addgene. Sequences of hairpin used for shRNAs were as follows: REV7 (5′-CCGGCCCTGATTCCAAGTGCTCTTACTCGAGTAAGAGCACTTGGAATCAGGGTTTTT), scramble (5′-CCTAAGGTTAAGTCGCCCTCGCTCGAGCGAGGG-CGACTTAACCTTAGG).

### Protein purification, GST pull down assay, immunoprecipitation assay and immunoblotting

Protein purification from *Escherichia coli* was done as described previously (22,26). Immunoprecipitation (IP) was done as described previously (27,28). Briefly, 48 h after transfection (for FH-REV3L) or 24 h after transfection, the cells were harvested, frozen in liquid nitrogen and stored at −80°C. Each cell pellet was suspended with 300 μl of 0.5B (500 mM KCl, 20 mM Tris–HCl [pH 8.0], 5 mM MgCl_2_, 10% glycerol, 1 mM PMSF, 0.1% Tween 20, 10 mM β-mercaptoethanol), frozen in liquid nitrogen, thawed on ice and sonicated. After centrifugation 900 μl of 2B (40 mM Tris–HCl [pH 8.0], 20% glycerol, 0.4 mM EDTA, 0.2% Tween 20) was added to the supernatant and incubated with 10 μl of anti-FLAG (Sigma) or GFP agarose (MBL) for 4 h at 4°C. The bound proteins were washed with 700 μl of 0.1B three times and eluted with 30 μl of 2× sodium dodecylsulphate (SDS) loading buffer (100 mM Tris–HCl [pH 6.8], 4% SDS, 0.2% bromophenol blue, 20% glycerol, 200 mM dithiothreitol (DTT). These samples were separated by polyacrylamide gel electrophoresis, transferred to a membrane and detected with the indicated antibodies and ECL reagents (GE Healthcare).

GST-fusion protein and His-REV7 and Glutathione-Sepharose 4B (GS4B) beads (GE Healthcare) were incubated at 4°C for 4 h in 500 μl of 0.1B. The beads were washed three times with 0.1B and eluted with 30 μl of 2× SDS loading buffer (100 mM Tris–HCl [pH 6.8], 4% SDS, 0.2% bromophenol blue, 20% glycerol, 200 mM DTT).

### Gene expression analysis, RNA-seq library construction and sequencing

Expression of the recombinant human *REV3L* was established using a human-specific Taqman assay (Life Technologies) at the exon 14–15 boundary: Ex14Fwd: 5′-CACCTGGCCTTAGCCCATTAT-3′, Ex15Rev: 5′-CTCTTCTAAGAGTGTCAGTATTACTTCCTTTC-3′ Probe: FAM-MGB-5′-CAACAGAACCAAAAACA-3′. To compare the recombinant expression to that of endogenous mouse *Rev3L*, we made a set of primers and a probe that would recognize both human and mouse *Rev3L* and would not amplify any knockout transcript. The primers/probe were at the exon 26/27 boundary: Ex26Fwd: 5′-GTGAATGATACCAAGAAATGGGG-3′; Ex27Rev: 5′-GTGAATGATACCAAGAAATGGGG-3′; Probe: FAM-MGB-5′ 5′-TACTGACAGTA-TGTTTGT-3′. Expression of the endogenous human *REV7* was established using MAD2L2 TaqMan Gene Expression Assay (Life Technologies : Catalog #: 4331182). Assay ID was Hs01057448_m1. Gene expression with mouse or human GAPDH as an expression control was measured on an Applied Biosystems 7900HT Fast Real-Time PCR System.

For RNA sequencing analysis, 100 ng of total RNA samples were converted to cDNA using a NuGEN Ovation RNA-Seq System v2 according to the manufacturer's protocol (NuGEN, San Carlos, CA, USA). NuGEN-amplified double-stranded cDNAs were fragmented into ∼180 bp using a Covaris system (Covaris, Woburn, MA, USA). Fragmented cDNAs were run on a SPRI-TE library construction system (Beckman Coulter, Fullerton, CA, USA), and during the adaptor ligation step, uniquely indexed NEXTflex adapters (Bioo Scientific, Austin, TX, USA) were used for each of the samples to allow for multiplexing. Adapter-ligated libraries were enriched by PCR using a KAPA library amplification kit (KAPA biosystems, Wilmington, MA) (1 cycle at 98°C for 45 s; 7 cycles of 98°C for 15 s, 65°C for 30 s and 72°C for 30 s; 1 cycle at 72°C for 1 min) and purified with AmpureXP beads (Beckman Coulter). The purified libraries were quantified using a Kapa library quantification kit (KAPA biosystems). The libraries were loaded on cBot (Illumina, San Diego, CA, USA) at final concentration of 10 pM to perform cluster generation, followed by 2 × 76 bp sequencing on HiSeq 2000 (Illumina).

### DNA damage sensitivity and micronuclei formation in *Rev3L* MEFs

*Rev3L* proficient (*Rev3L*^+/−^) and deficient (*Rev3L*^−/−^) T-antigen immortalized mouse embryonic fibroblasts (MEFs) were as described (11). Two parental *Rev3L*^−/−^ cell lines were used, cl 5 and cl 11. One parental *Rev3L*^−/−^ cell line was used, clone 5, for the 4A mutants, and two parental cell lines, clones 5 and 11 were used for the 2A mutants. The pOZN-REV3L vectors, were infected into these cell lines and subclones were selected (Lange *et* *al*., submitted). Methods of cell infection and selecting stable cell lines were done as described previously (29).

To analyze sensitivity to chemical DNA damaging agents, the immortalized MEFs were plated into white 96-well plates (5000 cells/well). The next day, various concentrations of cisplatin (Sigma) was added to the wells and the cells were incubated for 48 h. Then the cells were lysed in a reagent that luminesces in the presence of ATP (ATPLite One Step, Perkin Elmer), then luminescence was measured using a plate reader (Biotek Synergy II) and normalized to undamaged control. To analyze sensitivity of immortalized MEFs to ultraviolet C 254 nm peak (UVC) radiation, 3 × 10^5^ cells were pelleted and resuspended in 300 μl of phosphate-buffered saline. Three 100 μl drops were placed into the middle of a plastic dish and 10 μl aliquots from each were plated into 100 μl of growth media in a white 96-well plate after 0, 2.5, 5, 7.5, 10, 15 or 20 J/m^2^ UVC radiation at a dose rate of 0.4 J/m^2^/s. Forty-eight hours after irradiation, viability was measured as above.

To assess genomic instability in the *Rev3L*-deficient and complemented MEFs, micronuclei formation was measured. The MEFs were plated into an 8-well chamber slide and the following day, were fixed with paraformaldehyde and stained with DAPI. Photographs of the immunofluorescence were taken with a digital camera attached to a Leica DMI6000B microscope. Micronuclei were counted as small, separate DAPI stained foci adjacent to DAPI-stained nuclei. Statistical analysis was done using unpaired *t*-test with Welch's correction (*P* < 0.05).

### Quantification of REV7 protein

Cells were harvested 48 h after transfection (pEGFP vector). 1.46 × 10^7^ cells were lysed in 500 μl lysis buffer. One hundred microliter lysed sample (2.9 × 10^6^ cells) was mixed with 25 μl 5× SDS loading buffer for a total of 125 μl, yielding 2.3 × 10^5^ cells per 10 μl. The concentration of purified His-REV7 protein from *E. coli* was measured by the Bradford method. These samples were separated by polyacrylamide gel electrophoresis, transferred to a membrane and detected with an antibody against REV7. The signal intensity was calculated by ImageJ64 software. The signals of 10 and 5 μl whole cell extract indicated 5.4 and 2.7 ng, respectively, resulting in an estimate of ∼ 5.7 × 10^5^ REV7 molecules per 293T cell.

## RESULTS

### REV7 interacts with full length REV3L *in vivo*

**Figure 1.**
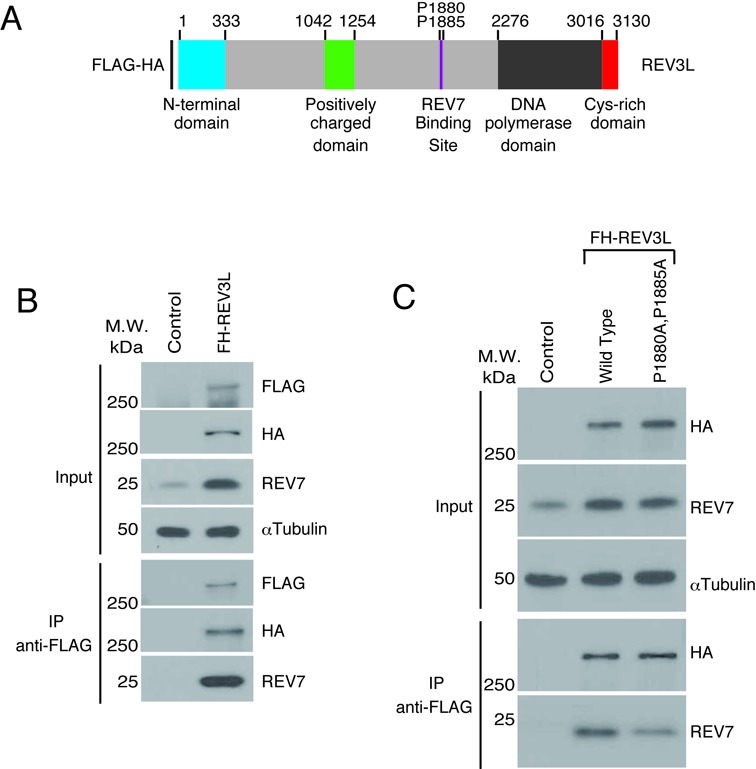
REV7 interacts with full-length REV3L *in vivo.* (**A**) Schematic of REV3L, showing the locations of the evolutionarily conserved N-terminal domain, the B-family DNA polymerase domain and the previously proposed REV7 binding site. An iron-sulfur cluster in the Cys-rich domain near the C-terminus provides a binding site for the POLD2 subunit. The position of FLAG and HA tags is shown. (**B**) Human 293T cells were transfected with either epitope-tagged REV3L (FH-REV3L) or Flag-HA empty vector (Control). Forty-eight hours after transfection cell lysates were made and used for immunoprecipitation with FLAG antibody beads. After electrophoretic transfer of proteins, the membrane was cut into three sections to separate proteins >250 kDa (FLAG), 37 to 250 kDa (HA and α-tubulin) and <37 kDa (REV7) and immunoblotted with the indicated antibodies. Results for the input and IP product after gel electrophoresis are shown. (**C**) FLAG-IP experiment similar to part A, using FH-REV3L, FH-REV3L mutant construct (P1880A, P1885A) and control empty vector.

Human REV7 is known to interact with short recombinant fragments of human REV3L (30–33) and with recombinant REV3L (17,34). We sought to determine the nature of the interaction between full-length human REV3L and REV7 *in vivo*. Human *REV3L* cDNA was inserted into a mammalian expression vector to express N-terminal FLAG-HA tagged REV3L (Figure 1A) from the EF1α promoter in a pCDH vector. Whole cell extracts from 293T cells were transiently transfected with pCDH/FH-REV3L or an empty vector control. Full-length recombinant REV3L was expressed and could be detected in cell extracts using FLAG and HA antibodies at the predicted molecular weight (∼350 kDa) (Figure 1B, ‘input’ lanes). Endogenous REV7 was also identified. IP of recombinant REV3L from cell extracts was carried out with FLAG antibody beads (35). Full length FH-REV3L could be detected by immunoblotting of the IP product when cells were transfected with pCDH/FH-REV3L but not with empty vector control (Figure 1B and Supplemental Figure S1A). Anti-FLAG immunoblotting of the FLAG IP product also detected lower molecular weight products (Supplemental Figure S1A, brackets), produced by premature translational truncation or proteolytic cleavage of full length REV3L (Supplemental Figure S1A). Endogenous REV7 co-immunoprecipitated with recombinant REV3L (but not with the control vector), demonstrating that full-length REV3L interacts with REV7 in human cells (Figure 1B). Expression of recombinant REV3L increased the abundance of endogenous REV7 in cells (Figure 1B), an observation that was explored in more detail below.

Mutation of prolines 1880 and 1885 to alanine in human REV3L has been shown to eliminate the interaction between REV7 and a recombinant fragment of REV3L (32). We attempted to abolish REV7-REV3L binding by introducing the P1880A and P1885A mutations (Figure 1A) into full-length REV3L. However, endogenous REV7 was still co-immunoprecipitated with this mutant REV3L (Figure 1C), suggesting the existence of an additional REV7 binding site in REV3L.

### Identification of a second REV7 binding site in human REV3L

**Figure 2.**
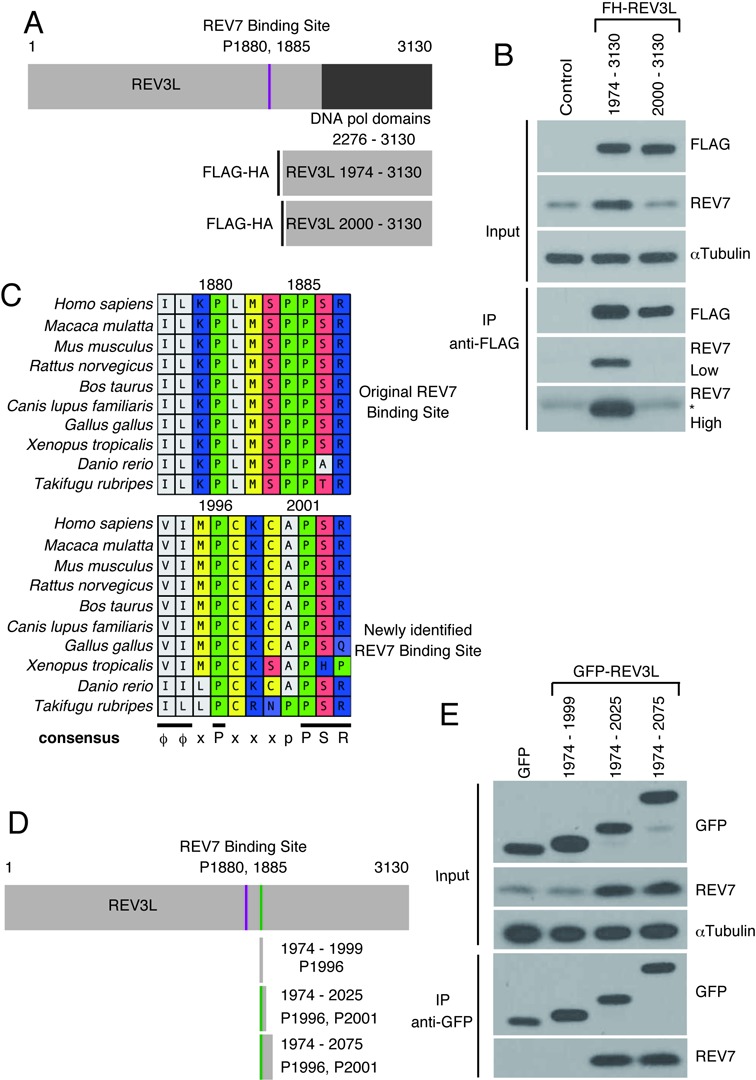
Identification of a novel *in vivo* REV7 binding region in REV3L. (**A**) Schematic showing vectors expressing FH-REV3L amino acid residues 1974–3130 and 2000–3130. (**B**) FLAG-IP of 293T cells transfected with FH-REV3L 1974–3130, FH-REV3L 2000–3130. The bottom panels show low intensity and high intensity exposures. IgG light chain migrating slightly above REV7 is indicated by the asterisk (*). (**C**) Sequence alignments of a region of REV3L, showing the original REV7 and newly identified REV7 binding sites, with the consensus sequence ϕϕxPxxxpPSR at the bottom. Numbers refer to human REV3L residues. (**D**) Schematic drawing showing locations of the REV3L fragments. The P1996/P2001 position is highlighted with a vertical bar (green in the online version). (**E**) Immunopurification of GFP fusion fragments of REV3L with anti-GFP agarose. Immunoblotting used anti-FLAG, anti-GFP, anti-REV7 and anti-αTubulin.

To explore this possibility, two FH-tagged REV3L fragments (amino acids 1974–3130 and 2000–3130) were expressed in cells (Figure 2A). Endogenous REV7 was immunoprecipitated with FH-REV3L 1974–3130. No interaction was detected with FH-REV3L 2000–3130, even with higher increased loading of protein or higher exposures (Figure 2B). This result suggests that a second REV7 binding site lies within the 1974–2000 amino acid region. Intriguingly, a sequence motif ϕϕxPxxxxPSR is present at residues 1993–2003 (where ϕ represents an aliphatic amino acid residue), similar to the known original REV7 binding site at amino acids 1877–1887 (Figure 2C). These sequences in vertebrate REV3L are consistent with the consensus of ϕϕxPxxxpP suggested for REV7 binding sites in various proteins by Hanafusa *et al.* (32). Both ϕϕxPxxxxPSR sequences are conserved in vertebrate REV3L proteins (Figure 2C). In the yeast *S.* *cerevisiae*, Rev7 binds yeast Rev3 somewhere in the region between residues 300 and 660 (19) but the precise binding site is unknown. A conserved consensus sequence similar to the motif observed in vertebrates (or even PxxxxP) is not apparent in Rev3 from fungi (Ascomycota), including *S. cerevisiae* and *Schizosaccharomyces pombe*.

A co-crystal structure shows that specific interactions occur between REV7 and the proline residues of a REV3L 1847–1898 peptide (33). Residues P1880 and P1885 are particu­larly important, as a double mutation of both of these Pro residues to Ala eliminates binding of the 1847–1898 peptide to REV7 (32). To test directly whether the region between REV3L residues 1974 and 2000 can also mediate binding of REV7 to REV3L, several GFP fusion frag­ments of REV3L were constructed and expressed in cells: GFP-REV3L 1974–1999, GFP-REV3L 1974–2025 and GFP-REV3L 1974–2075 (Figure 2D). After IP, endogenous REV7 was detected in association with GFP-REV3L 1974–2025 and GFP-REV3L 1974–2075, but not with GFP-REV3L 1974–1999 (Figure 2E). These data demonstrate that a 52-residue fragment of human REV3L containing only the second putative binding motif can bind REV7 in cells.

**Figure 3.**
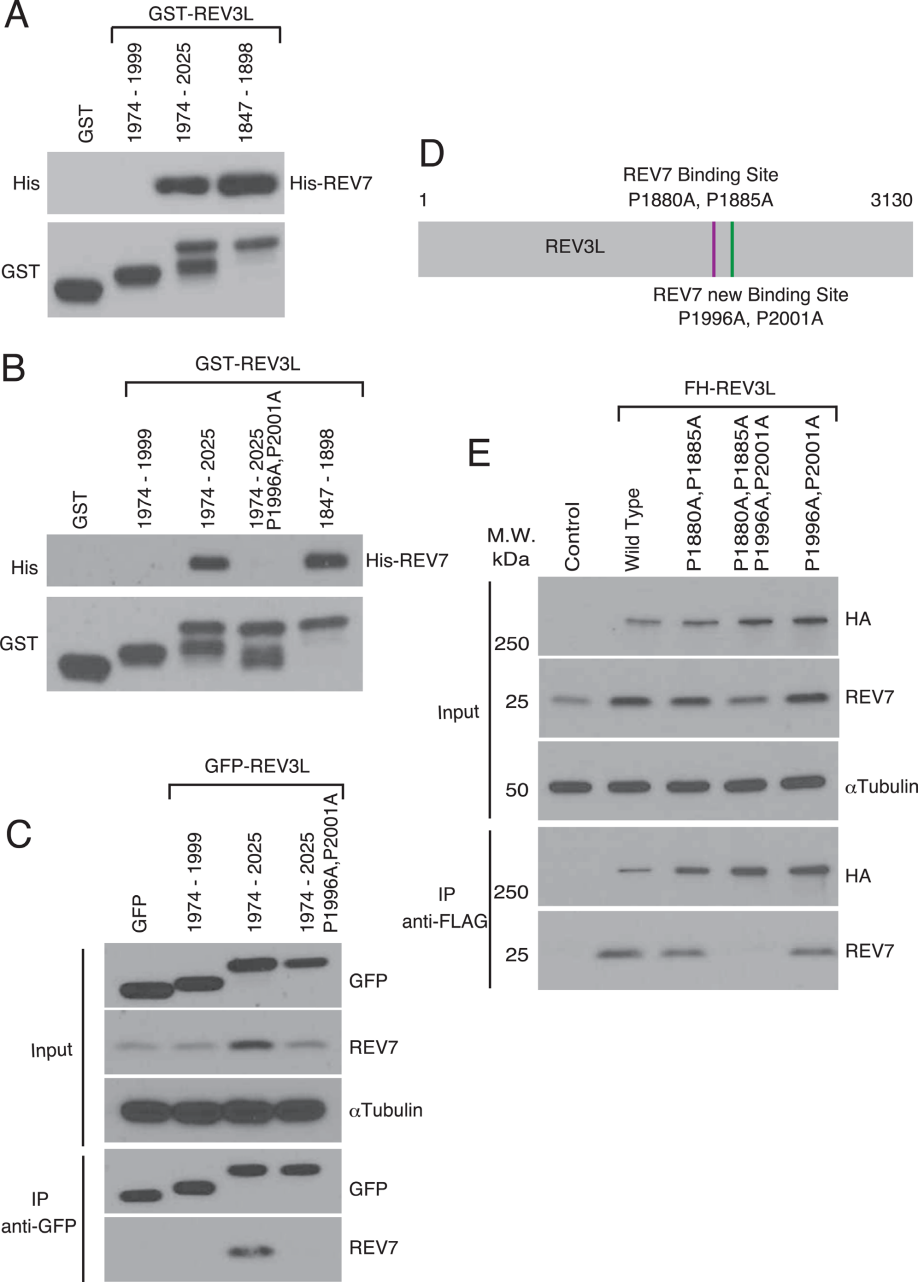
A novel REV7 binding site in REV3L. (**A**) *In vitro* association of REV7 with fragments of REV3L. GST fusion fragments of REV3L and His-REV7 fusion protein were purified from *Escherichia coli* (see Supplementary Figure S1B). These were used with glutathione beads for GST pulldown experiments. After electrophoresis, samples were immunoblotted with anti-His or anti-GST as indicated. The positive control REV3L 1847–1898 fragment included the original REV7 binding site (P1880, P1885); the negative control REV3L 1974–1999 fragment is unable to bind REV7 (Figure 2E). (**B**) *In vitro* GST pulldown of purified REV3L fragments (see Supplementary Figure S1C) containing the indicated amino acid changes. (**C**) GFP-REV3L fusion fragments (and mutant versions) were expressed in human 293T cells (Input) and then immunoprecipitated with anti-GFP (IP). Following gel electrophoresis, immunoblotting was performed with the indicated antibodies. (**D**) Schematic of REV7 binding sites in REV3L. (**E**) FLAG-IP assay of 293T cells transfected with FH-REV3L, FH-REV3L (P1880A, P1885A), FH-REV3L (P1880A, P1885A, P1996A, P2001A), FH-REV3L (P1996A, P2001A) or control empty vector. For immunoblotting, we used anti-HA, anti-REV7 and anti-αTubulin.

Experiments using GST-tagged purified fragments of REV3L further confirmed that this region (REV3L 1974–2025) binds directly to REV7 independently of the original REV7 binding site within human REV3L (Figure 3A and Supplemental Figure S1B). When the conserved prolines of the novel REV7 binding site were mutated (P1996A, P2001A), no His-REV7 binding was detectable *in vitro* after the GST pull-down experiment (Figure 3B and Supplemental Figure S1C). This demonstrates that these Pro residues in the ϕϕxPxxxxPSR motif must be maintained in order for the novel binding site to physically interact with REV7. We next tested the ability of this mutant peptide to bind endogenous REV7 in human cells. GFP-REV3L 1974–2025 (P1996A, P2001A) mutant vector was transfected into 293T cells. Subsequent IP showed that the mutant REV3L peptide does not bind REV7 *in vivo* (Figure 3C).

Although these experiments established a second REV7 binding site in REV3L in the region 1974–3130, the possibility remained that additional binding sites for REV7 might be present between REV3L amino acid residues 1–1973. To test this possibility, 293T cells were transfected with full length FH-REV3L or with mutant REV3L harboring mutations in either of the two REV7 binding sites or both binding sites (Figure 3D). Mutation of both REV7 binding sites in REV3L completely abolished the ability to bind endogenous REV7 (Figure 3E). However, mutations in either of the individual REV7 binding sites in REV3L still allowed for detectable REV7 binding as expected. These data suggest that only two REV7 binding sites exist in REV3L and that binding at both sites must be eliminated in order to abolish REV3L–REV7 interaction.

Expression of recombinant REV3L in cells increased the abundance of endogenous REV7 (Figures 1B, 2E and 3E). We noted that disruption of REV7-REV3L binding resulted in decreased endogenous REV7 and elevated REV3L protein levels compared to controls (Figure 3E). A recent study reported anaphase-promoting complex-dependent degradation of REV1 mediated through REV7 binding (36). We therefore investigated whether REV7 may also promote REV3L degradation. Expression of recombinant REV3L in 293T cells was increased by cotransfection with REV7 shRNA (Supplemental Figure S2A and B), suggesting that REV7 negatively regulates REV3L levels. Expression of FH-REV3L in the presence of proteasome inhibitor MG132 increased REV3L expression (Supplemental Figure S2C). Similar protection from degradation was seen in MG132-treated cells that express WT or a REV3L mutant version that abolishes REV7 binding (Supplemental Figure S2B).

We also investigated the impact of REV7 levels on transcription of *REV3L*, which is plausible because REV7 is known to bind the transcription factor ELK1 (37). We analyzed RNA-seq data, comparing cell lines that overexpress REV7 or were suppressed for REV7. The level of *REV3L* mRNA was independent of *REV7* mRNA level (Supplemental Figure S3). We also quantified *REV7* mRNA when REV3L was expressed ectopically (Supplemental Figure S4A). The amount of REV7 mRNA was also unchanged (Supplemental Figure S4B). These data suggest that although REV3L and REV7 protein levels are inversely correlated and that REV3L is stabilized by proteasome inhibition, REV7–REV3L interaction via the two domains studied here is not necessary for proteosomal-mediated degradation of REV3L.

### REV7 interaction with REV3L is needed to confer resistance to DNA damage

**Figure 4.**
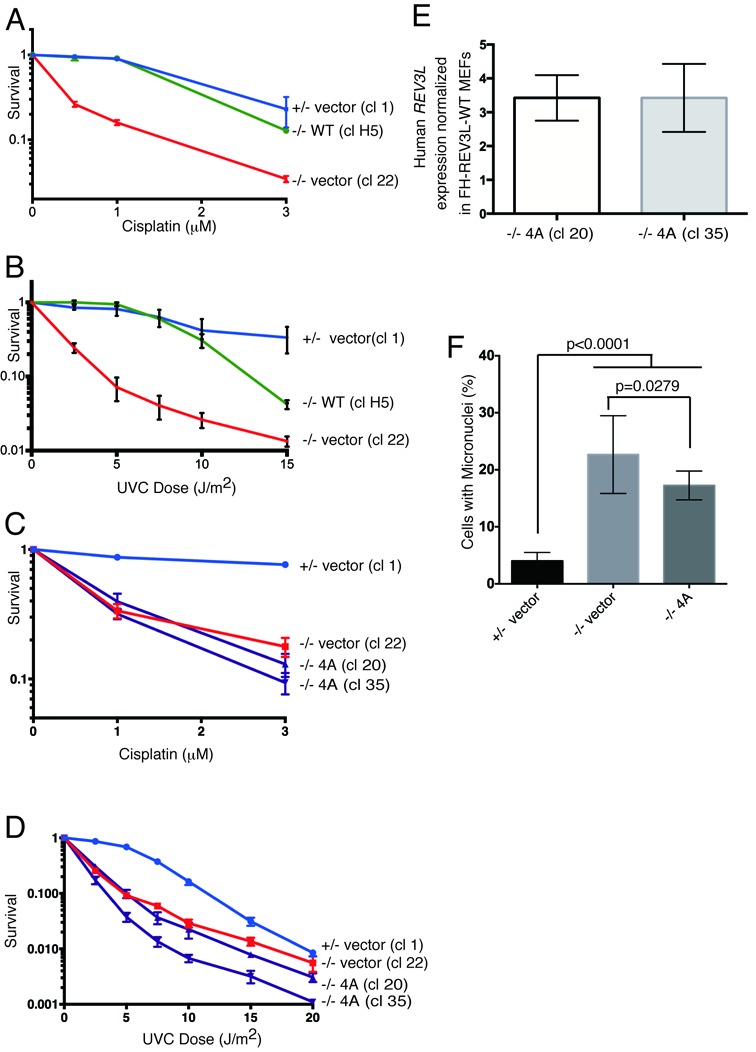
Both REV7 binding sites in REV3L are important for cellular resistance to DNA damage. (**A** and **B**) Expression of human REV3L rescues cisplatin and UVC sensitivity of *Rev3L*-null MEFs. The survival of three clones was monitored following cisplatin and UVC treatment. +/− vector (cl 1): a control *Rev3L*^+/−^ subclone containing pOZN-empty vector (square symbols, blue line). −/− vector (cl 22): a *Rev3L*^−/−^ knockout subclone containing pOZN-empty vector (triangle symbols, red line). −/− WT (cl H5) : a *Rev3L*^−/−^ subclone containing pOZN- WT REV3L expression vector (circle symbols, green line). Quantitative RT-PCR with primers specific to human *REV3L* verified that human *REV3L* was expressed in the MEFs. MEF cell line clones stably expressing the indicated REV3L constructs were plated and exposed to the indicated doses of cisplatin (**C**) or UVC (**D**). Cellular viability was measured 48 h later. +/− vector (cl 1): circle symbols, blue line. −/− vector (cl 22): square symbols, red line. −/− 4A (cl 20) and cl 35 : a *Rev3L*^−/−^ subclone containing pOZN-REV3L P1880A, P1885A, P1996A, P2001A expression vector (triangle symbols, purple line). (**E**) *Rev3L*-null MEFs stably expressed 4A-mutant *REV3L* at a ∼3-fold higher levels than *Rev3L*-null MEFs complemented with WT REV3L. *Gapdh* was used as an internal control. (**F**) REV3L mutants that impair REV7 binding are unable to rescue chromosomal instability. +/− vector (cl 1, 2, 14, 26): *Rev3L*^+/−^ subclones containing pOZN-empty vector; −/− vector (cl 2, 12, 20): *Rev3L*^−/−^ subclones containing pOZN-empty vector; −/− 4A (cl 20, 35): *Rev3L*^−/−^ subclones containing pOZN-4A binding mutant. Infections of clones were done independently and repeated on different days. Cells were fixed and stained with DAPI and micronuclei were enumerated for at least three independent experiments per clone. Data represent mean ± SEM. (*) *P* < 0.05 by unpaired *t*-test.

To determine the biological consequences of a lack of REV3L–REV7 interaction within mammalian pol ζ, we used *Rev3L* MEF cell lines (11). Subclones of null and heterozygous MEFs expressing human *REV3L* cDNA were selected and assayed for sensitivity to cisplatin and UVC radiation. Complementation of *Rev3L*^−/−^ cells with a retrovirus expressing human REV3L cDNA restored DNA damage resistance to the levels of *Rev3L*^+/−^ control cells (Figure 4A and B).

We isolated *Rev3L*^−/−^ cell clones stably expressing a mutant version of REV3L that was unable to bind to REV7 (P1880A, 1885A, P1996A, P2001A: ‘4A mutant’). The 4A mutant cDNA was unable to rescue the cisplatin or UVC hypersensitivity of *Rev3L^−/−^* MEFs (Figure 4C and D, Supplemental Figure S5A), even when expressed at a level three to four times higher than cells complemented with WT REV3L (Figure 4E). Furthermore 4A mutant cDNA could not rescue the elevated level of spontaneous micronuclei that indicates chromosomal breakage in the *Rev3L*^−/−^ cells (Figure 4F, Supplemental Figure S5E and F). Surprisingly, REV3L mutant cDNA with mutations of either binding site alone, either the original REV7 binding site (P1880A, 1885A; ‘O2A mutant’) or the newly identified REV7 binding site (P1996A, P2001A; ‘N2A mutant’), could not rescue sensitivity to DNA damage (Supplemental Figure S5B and C). cDNAs expressing either 2A mutant form of REV3L also did not rescue the high frequency of micronuclei (Supplemental Figure S5E and G), even though the mutant cDNAs were expressed at a level 3–50 times higher than cells complemented with WT REV3L (Supplemental Figure S5D). These data show that both REV7 binding sites in mammalian REV3L are important for resistance to DNA damage-induced cell death and promotion of genome stability.

## DISCUSSION

**Figure 5.**
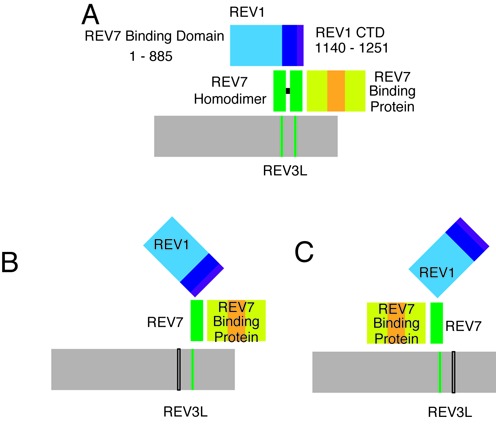
Model employing for promotion of functional interactions of REV7 by two binding sites in REV3L. (**A**) Two binding sites for REV7 on REV3L (vertical bars) would stabilize a homodimer of REV7. This would be functionally useful because REV7 has multiple binding partners (represented generically as ‘REV7 binding protein’ and REV7 interacts with two sites on REV1. In addition, some REV7 binding proteins may bind to the same region of REV7, so that two molecules of REV7 would be required to facilitate simultaneous interactions with multiple proteins. (**B** and **C**) Inactivation of one of the REV7 binding sites (black open bars) would still allow a monomer of REV7 to bind REV3L, but prevent the stable interaction with REV1 or other REV7 binding proteins. CTD, C-terminal domain. Black horizontal bar, Dimerization.

This study provides evidence for two REV7 binding sites within human REV3L. Both REV7 binding sites on human REV3L are necessary in order to confer resistance to DNA damage and ward off genomic instability. The discovery of two REV7 binding sites is likely to be relevant to the observation that REV7 can form a homodimer (31). Although a REV–REV7 interaction can occur, it is relatively weak (32). By simultaneous interaction with two adjacent sites on REV3L, the dimerization of REV7 would be favored (Figure 5). A dimer of REV7 bound to REV3L could have considerable utility, allowing interaction with the many potential and relevant REV7 binding partners. For example, REV7 binds to REV1 (31,38) by interaction near the C-terminus (39,40) and to an additional domain in the N-terminal half (36). REV7/MAD2L2 also has numerous other known protein binding partners, including the CDH1 and CDC20 proteins (41,42) that operate in the mitotic checkpoint, as well as ELK1 (37), TCF4 (43), PRCC (44) and HCCA2 (45). Moreover, REV7 functions in epigenetic reprogramming by interacting with both G9a and GLP methyl­transferases (46). Binding of two molecules of REV7 to REV3L would provide multiple interfaces for the implementation of pol ζ in different chromosomal contexts. This additional level of complexity and regulation is not found in yeast pol ζ, which appears to contain a single Rev7 subunit (19).

Although REV7 and REV3L interact together in mammalian pol ζ, evidence is accumulating that the proteins also have functions independent of one another. The REV7 gene name *MAD2L2* reflects its similarity to MAD2 (Mitotic Arrest Deficient 2). REV7 protein is 4000 times more abundant than REV3L in *Xenopus* oocytes (47). We confirmed that REV7 is abundant by quantifying the level of endogenous REV7 in 293T cells (5.7 × 10^5^ molecules per cell) (Supplemental Figure S4C). As a result, REV7 is often found in complexes with other proteins (37,41–45,48), separately from pol ζ.

Moreover, disruption of *Rev7* in the mouse has very different consequences from disruption of mouse *Rev3L*. Inactivation of *Rev3L* in mice is incompatible with viability (5). Deletion of *Rev3L* from primary MEFs rapidly leads to senescence, unless cells are immortalized with SV40 T-antigen (11). In contrast, no obvious problems with proliferation were observed in cells of *Rev7*-disrupted mouse embryos (46,49,50) or in primary MEFs derived from them (46,50). Moreover, from embryonic days E8.5 to E13.5, *Rev7*^−/−^ embryos are present at a normal mendelian ratio, whereas no viable *Rev3L*^−/−^ embryos have ever been detected by the end of E13.5 in numerous experiments (5). Some *Rev7*^−/−^ mice are born (approximately one-third of the expected mendelian genotype ratio). These have a congenital defect in male spermatagonia after birth due to loss of primordial germ cells. The phenotypes of *Rev7*-null mice more closely resemble mice with defects in cell cycle regulation or particular stress responses (49).

Because REV3L disruption has much more severe consequences for cell proliferation than REV7 disruption, it appears that there is a REV7-independent role for REV3L in embryogenesis and normal cell proliferation. However, we show here that binding of REV3L to two molecules of REV7 is required for maintaining resistance to DNA damaging agents and for limiting spontaneous chromosome breaks in immortalized cells. The present study therefore reveals a separation of function within pol ζ, where both REV3L and REV7 are needed for damage resistance functions, even though REV7 is not necessary for some critical proliferation-based functions.

## SUPPLEMENTARY DATA

Supplementary Data are available at NAR Online.

SUPPLEMENTARY DATA
