# Supplementary material for: REV7 is essential for DNA damage tolerance via two REV3L binding sites in mammalian DNA polymerase ζ
Source: Nucleic Acids Res. 2015 Jan 7;43(2):1000–11. doi: 10.1093/nar/gku1385 (PMC4333420; doi:10.1093/nar/gku1385)
Supplement: SUPPLEMENTARY DATA [file supp_43_2_1000__index.html]

REV7 is essential for DNA damage tolerance via two REV3L binding sites in mammalian DNA polymerase ζ — SUPPLEMENTARY DATA 

# REV7 is essential for DNA damage tolerance via two REV3L binding sites in mammalian DNA polymerase ζ

## SUPPLEMENTARY DATA

**Files in this Data Supplement:**

- Supplementary Figures Captions
- Fig S1
- Fig S2
- Fig S3
- Fig S4
- Fig S5
