# Supplementary material for: REV7 is essential for DNA damage tolerance via two REV3L binding sites in mammalian DNA polymerase ζ
Source: Nucleic Acids Res. 2015 Jan 7;43(2):1000–11. doi: 10.1093/nar/gku1385 (PMC4333420; doi:10.1093/nar/gku1385)
Supplement: SUPPLEMENTARY DATA [file supp_gku1385_nar-03281-d-2014-File007.docx]

**Supplemental Figure 1. Purified recombinant REV7 and GST fusion REV3L fragments**

**(A)** Full membrane of the anti-FLAG immunoblot for the IP anti-FLAG gel segment designated in Figure 1B (5^th^ segment from the top). Cell extracts transfected with empty control vector or FH-REV3L were subjected to FLAG IP before immunoblotting. The asterisk (*) in the control lane marks a short polypeptide produced by the empty vector. The bracket at right indicates the position of truncated FLAG-HA-tagged products of REV3L expression. **(B)** GST fusion fragments of REV3L, and His-REV7 used for Figure 3A. were expressed in *E. coli*, purified, and stained with Coomassie brilliant blue. **(C)** GST fusion fragments of mutated REV3L as indicated, and His-REV7, used for Figure 3B.

**Supplemental Figure 2. REV7 does not control the REV3L protein level through proteasome dependent degradation.** (**A**) FH-REV3L or empty vector was co-transfected with shREV7 plasmid or shScramble control into 293T cells and whole cell extract was immunoblotted 48 h later. Full length FH-REV3L is indicated by “REV3L” and an arrow, and the REV3L* indicates a degraded or prematurely truncated form of REV3L. (**B**) 293T cells were transfected with FH-REV3L wild-type, FH-REV3L P1880A, P1885A, P1996A, P2001A or empty vector. MG132 or DMSO was added to 293T cells 36 h after transfection and cells were harvested 48 h after transfection and immunoblotted. (**C**) FH-REV3L, FH-REV3L P1880A, P1885A, P1996A, P2001A or empty vector was co-transfected with shREV7 plasmid or shScramble into 293T cells and lysates were immunoblotted 48 h later. For immunoblotting, we used anti-HA, anti-FLAG, anti-REV7 and anti-αTubulin.

**Supplemental Figure 3.** The relative amounts of *REV7* and *REV3L* expression were measured by RNA-sequencing when REV7 was stably expressed or depleted in HeLa S3 cells. Three independent experiments were performed and *GAPDH* expression was as an internal control.

**Supplemental Figure 4. The relationship between REV7 and REV3L expression level.**

(**A**) REV7 accumulates in cells when REV3L was expressed. 293T cells with FH-REV3L wild-type or empty vector were transfected and cells were harvested 48 h and immunoblotted.

(**B**) *REV7* mRNA levels do not increase when REV3L was expressed. 293T cells with FH-REV3L wild-type or empty vector were transfected and cells were harvested 48 h and a q-PCR assay was conducted. Expression normalized to *GAPDH*. (**C**) The amount of REV7 in the cell. His-REV7 fusion protein purified from *E. coli.* 293T cells with GFP empty vector were transfected and cells were harvested 48 h later and immunoblotted with the indicated antibodies.

**Supplemental Figure 5. Mutation of both binding sites resulted in sensitivity to DNA damage. (A)** Expression of mutant REV3L 4A mutant does not rescue cisplatin and UVC sensitivity of *Rev3L*-null MEFs. Expression of individual 2A mutant REV7 binding sites also cannot rescue cisplatin **(B)** and UVC sensitivity **(C)**. The survival of six cultures was monitored following cisplatin and UVC treatment.(+/-) vector cl 1: a *Rev3L*^+/-^ subclone containing pOZN-empty vector. (-/-) vector cl 22: a *Rev3L*^-/-^ subclone containing pOZN-empty vector. (-/-) 5 N2A1 and (-/-) 11 N2A1: *Rev3L*^-/-^ pre-clonal cell lines containing pOZN-REV3L P1996A, P2001A expression vector. (-/-) 5 O2A1 and (-/-) 11 O2A1: *Rev3L*^-/-^ pre-clonal cell lines containing pOZN-REV3L P1880A, P1885A expression vector. 5 N2A1, (-/-) 11 N2A1, (-/-) 5 O2A1 and (-/-) 11 O2A1 were infected by viruses that were made on a different day. **(D)** The 2A-mutant complemented *Rev3L*-null MEFs expressed *REV3L* at higher levels than the control *Rev3L*-deficient MEFs that were complemented with wild-type REV3L. **(E)** Expression of mutant *REV3L* lacking individual REV7 binding sites does not rescue chromosomal instability. (+/-) vector (cl 1, 2, 14, cl 26): *Rev3L*^+/-^ subclones containing pOZN-empty vector; (-/-) vector (cl 2, 12, 22): *Rev3L*^-/-^ subclones containing pOZN-empty vector; (-/-) O2A or N2A: *Rev3L*^+/-^ subclones containing pOZN-2A mutant vector that were independently infected on different days into two different parental clones (-/- clones 5 and 11). Data represent mean ± SEM. (*) p<0.05 by unpaired t-test. **(F and G)** Scoring of individual clones or pre-clonal cell populations used to generate the data in Figure 4F and Supplemental Figure 4E respectively.
